# Exploring the relationship between polio type 2 serum neutralizing antibodies and intestinal immunity using data from two randomized controlled trials of new bOPV-IPV immunization schedules

**DOI:** 10.1016/j.vaccine.2017.11.006

**Published:** 2017-12-19

**Authors:** Ananda S. Bandyopadhyay, Edwin J. Asturias, Miguel O'Ryan, M. Steven Oberste, William Weldon, Ralf Clemens, Ricardo Rüttimann, John F. Modlin, Chris Gast

**Affiliations:** a Bill & Melinda Gates Foundation, Seattle, WA, USA; b Department of Pediatrics, University of Colorado School of Medicine, Aurora, CO, USA; c Center for Global Health and Department of Epidemiology, Colorado School of Public Health, Aurora, CO, USA; d Millennium Institute of Immunology and Immunotherapy, Faculty of Medicine, University of Chile, Santiago, Chile; e Centers for Disease Control and Prevention, Atlanta, GA, USA; f Global Research in Infectious Diseases (GRID), Rio de Janeiro, Brazil; g Fighting Infectious Diseases in Emerging Countries (FIDEC), Miami, FL, USA; h Fred Hutchinson Cancer Research Center, Seattle, WA, USA

**Keywords:** Poliovirus, Vaccination, Humoral immunity, Intestinal immunity, Endgame

## Abstract

**Background:**

Inactivated polio vaccine (IPV) is now the only source of routine type 2 protection. The relationship, if any, between vaccine-induced type 2 humoral and intestinal immunity is poorly understood.

**Methods:**

Two clinical trials in five Latin American countries of mixed or sequential bOPV-IPV schedules in 1640 infants provided data on serum neutralizing antibodies (NAb) and intestinal immunity, assessed as viral shedding following oral mOPV2 challenge. Analyses with generalized additive and quantile regression models examined the relationships between prechallenge NAb titers and proportion, duration and titers (magnitude) of viral shedding.

**Results:**

We found a statistically significant (p < .0001) but weak relationship between NAb titer at the time of mOPV2 challenge and the Shedding Index Endpoint, the mean log_10_ stool viral titer over 4 post-challenge assessments. Day 28 post-challenge shedding was 13.4% (8.1%, 18.8%) lower and the Day 21 post-challenge median titer of shed virus was 3.10 log_10_ (2.21, 3.98) lower for subjects with NAb titers at the ULOQ as compared with LLOQ on day of challenge. Overall, there was a weak but significant negative relationship, with high NAb titers associated with lower rates of viral shedding, an effect supported by subset analysis to elucidate between-country differences.

**Conclusions:**

Taken alone, the weak association between pre-challenge NAb titers following IPV or mixed/sequential bOPV/IPV immunization and differences in intestinal immunity is insufficient to predict polio type 2 intestinal immunity; even very high titers may not preclude viral shedding. Further research is needed to identify predictive markers of intestinal immunity in the context of global OPV cessation and IPV-only immunization.

## Highlights

• Bivalent Oral Polio Vaccine (bOPV) without type 2 poliovirus has replaced trivalent OPV globally.
• Inactivated polio vaccine is now the only elective source of protection against type 2 poliovirus.
• We examined the relationship of humoral and intestinal type 2 immunity from new IPV-bOPV schedules.
• High neutralizing antibody titers were weakly associated with lower type 2 shedding.

## 1 Introduction

The Global Polio Eradication Initiative is on the verge of achieving its goal of interruption of wild polio virus (WPV) transmission [1]. To accelerate the progress made and to ensure transmission of all polioviruses is effectively interrupted, the Polio Eradication & Endgame Strategic Plan recommended the adoption of new polio vaccination schedules worldwide [2]. The first step was a switch in April 2016 from trivalent oral poliovirus vaccine (tOPV) to bivalent OPV (bOPV, types 1 and 3) in primary immunization series accompanied by introduction of at least one dose of inactivated poliovirus vaccine (IPV) in OPV-using countries.

Both humoral and mucosal immunity are important for polio eradication strategies [3]. Humoral immunity, measured as neutralizing antibody titers in serum post-vaccination, is an indicator of long-lasting individual protection against paralysis caused by poliovirus. Intestinal immunity, which develops after mucosal infection with wild or vaccine polioviruses and provides temporary protection against person-to-person transmission, is more difficult to assess [3], [4], [5], [6]. Typically, pharyngeal or intestinal mucosal immunity are measured as the extent of viral excretion following an oral challenge with live attenuated vaccine. In settings of poor hygiene and sanitation, intestinal mucosal immunity is considered more relevant than pharyngeal immunity, and therefore most studies have focused on intestinal excretion of challenge viruses [3], [7]. Alternative methods to assess intestinal mucosal immunity, such as directly measuring specific antibodies in excreta or circulating antigen-specific antigen-secreting cells (ASC) that express receptors for mucosal homing [5], [6], [8], are under evaluation with the promise of potentially replacing the accepted method of measuring shedding in the future.

IPV is now the only routinely available source of polio type 2 immunity. Although the per-dose effectiveness of IPV in producing humoral immunity as measured by seroconversion and neutralizing antibody (NAb) titers has been well established, its relationship to primary intestinal mucosal immunogenicity is limited and less clearly understood. Of interest, in relation to the global switch from tOPV to bOPV is the impact on type 2 intestinal immunogenicity from one or more dose(s) of IPV. Recent randomized controlled trials exploring bOPV–IPV schedules followed by mOPV2 challenge have concluded that although regimens including IPV reduce the duration and titer of viral shedding, they tend to be associated with limited overall impact on virus shedding, particularly during the time that virus excretion peaks, at around 7 days following oral challenge [9], [10], [11].

As there are often significant variations in levels of serum NAbs within vaccination regimens, we used data on polio type 2 circulating antibodies and virus excretion dynamics obtained from recent randomized controlled trials conducted in Latin America to directly explore a potential relationship between individual pre-challenge serum NAb levels and intestinal immunity that should add value to the evidence base on the new schedules of polio vaccination.

## 2 Materials and methods

Data were derived from two recently published randomized controlled trials performed in 2013–2014: study IPV001, performed in Colombia, the Dominican Republic, Guatemala and Panama, and registered on clinicatrials.gov as NCT01831050 [11], and study IPV002, performed in Chile and registered as NCT01841671 [9]. These were the only trials that met the three criteria we considered important to simulate an OPV2 cessation era for our analysis: (1) mixed or sequential primary series of IPV and bOPV; (2) mOPV2 used as a challenge vaccine to elicit type 2 excretion dynamics; (3) assessment of both duration and extent of viral shedding for 28 days following oral challenge as well as proportion of the challenged population shedding virus. Both trials were approved by all relevant national and institutional ethical bodies. For IPV001 the Centro de Estudios en Infectologia Pediatrica, Cali, Colombia; Hospital Maternidad Nuestra Señora de la Altagraci, Santo Domingo, Dominican Republic; Hospital Roosevelt Guatemala, Guatemala City, Guatemala; and Hospital del Niño, Panama City, Panama, and the Colorado Multiple Institutional Review Board and the Western Institutional Review Board, and for IPV002 the Faculty of Medicine at the University of Chile, the Servicio de Salud Metropolitano Norte, and the Servicio de Salud Metropolitano Sur, all located in Santiago, Chile. For our analysis, we combined data from all subjects who received bOPV and or IPV in the primary vaccination series from IPV001 and IPV002. In IPV001, data for IPV from three different manufacturers were combined within vaccination regimen, wherever relevant, and additional data for some subjects who received only three doses of tOPV were included as controls.

The primary endpoint used for this analysis was a shedding index endpoint (SIE) [9], [11], computed for each subject as the average of the titers of shed virus in stool samples (log_10_ CCID_50_/g) collected on days 7, 14, 21, and 28 post-mOPV2 challenge. Viral titers were assessed in stool samples that had detectable OPV using RT-PCR, with a viral titer of 0 recorded for samples negative for OPV. Defined in this way, the SIE captures both the magnitude and duration of viral shedding. Also measured were the titers of serum neutralizing antibody (NAbs) immediately prior to mOPV2 challenge. All subjects from both studies with sufficient data available to compute the SIE and pre-challenge NAbs were used for this analysis. Both the SIE and NAb titer values are subject to censoring at their respective lower and upper limits of quantitation (2.5 and 10.5 for NAbs [log_2_], 2.75 and 8.25 [log_10_] for SIE) as the assay workflows were set up for specific ranges of dilutions and values outside of these ranges were not captured, resulting in the described LLOQ and ULOQ. If a positive sample had a titer below the lower limit of quantitation (LLOQ), this limit was used for the data point. All other limits of quantitation were used as data points for values exceeding them.

### 2.1 Statistical methods

Regression models were fitted to endpoints using NAb titer and/or nominal stool collection day as predictors to describe the relationship between the likelihood and extent of viral shedding as a function of NAbs on day of challenge. Shedding positivity at each day post-challenge was modelled using an additive generalized linear model (GAM), implemented with the mgcv package [12] for R [13], using a binomial error structure and separate smooth functions of NAb titers for each post-challenge collection day, in addition to a model which also incorporates an interaction between NAb titers and (numeric, continuous) post-challenge collection day. The smoother basis dimension and model structure was selected via analysis of deviance for nested models, and Akaike’s Information Criterion (AIC) elsewhere [14]. The quality of model fit is described with the area under the curve (AUC) of the receiver operating characteristic (ROC) curve.

The titer of shed virus at each post-challenge day was modelled using median regression, implemented in the quantreg package for R [15], and utilizing a cubic spline basis for the NAb predictor interacted with post-challenge collection day as a factor variable. The basis dimension and model structure was selected via Wald tests for nested models [16], and AIC elsewhere. The SIE was modelled in the same manner as titer of shed virus, with omission of the factor for day, and 4 degrees of freedom for the spline, selected via Wald tests for nested models, and AIC elsewhere. The quality of model fit is described by a goodness of fit measure for quantile regression [17], and expressed as percent reduction in unexplained variation by a more complex model relative to a simpler model.

To evaluate inter-country differences in the relationship between pre-challenge type 2 serum NAbs and viral shedding, due, perhaps, to different levels of passive type 2 exposure in the different study locations, a subset analysis of IPV001 data only was performed. IPV002 data were excluded to avoid confounding of country with regimen and timing; as randomization in IPV001 was balanced across country, the influence of regimen is not confounded with country in this subset. For all models, the NAb predictor was used in two different methods. The first considered the observed NAbs, using the limits of quantitation as observed data. The second method involved posing a pair of models for values above and below the limit of quantitation, respectively, as a sensitivity measure for the influence of this censoring. This second method involved repeatedly simulating the censored values from the models, re-fitting the regression model while reusing the observed (uncensored) data, and aggregating the results. For the lower limit of quantitation, it was assumed that NAb titers were uniformly distributed between 0 and the lower limit (2.5 log_2_). For the upper limit of quantitation (ULOQ), a shifted exponential model was assumed, with shift equal to the upper limit (10.5 log_2_) and a rate of 0.7. The rate was selected by manually tuning the resulting distribution to align with Sabin-2 NAb data from IPV vaccinees obtained from an assay with a higher ULOQ used in another study [18]. The models incorporating simulations for censored values were used to obtain point estimates and corresponding two-sided 95% confidence intervals.

## 3 Results

A total of 1640 subjects who did not receive any type 2 containing OPV before the mOPV2 challenge were available for analysis across a variety of bOPV and IPV regimens (Table 1), with an additional 81 subjects from study IPV001 who received three doses of tOPV and served as the gold standard control.

**Table 1:** Number of subjects available for analysis from studies with serotype 2 baseline immunogenicity measures and pre-challenge stool viral shedding quantities, by regimen, for studies IPV001 [11] and IPV002 [9]. All subjects were challenged with mOPV2 4 weeks following final vaccination, except for IPV001 Group 2, who were challenged 26 weeks following their last vaccination.

Study	Group	Regimen	Schedule	N‡	Seroprotection rate*	Median NAb titre (log_2_)*	Pre-challenge shedding positivity**	Median log_10_ viral titer among shedders**
IPV001	1	bOPV-bOPV-bOPV	6-10-14 wks	187	113/209 (54.1%)	3.2	1/18 (5.6%)	3.2
	2	bOPV-bOPV-bOPV	6-10-14 wks	179	124/210 (59.0%)	3.2	0/13 (0.0%)	N/A
	4/6/8†	bOPV-bOPV-bOPV + IPV	6-10-14 wks 14 wks	274	175/310 (56.5%)	3.5	2/28 (7.1%)	3.8
	5/7/9†	bOPV-bOPV-bOPV + IPV-IPV	6-10-14 wks 14-36 wks	519	327/590 (55.4%)	3.2	1/63 (1.6%)	4.1
	3	tOPV-tOPV-tOPV	6-10-14 wks	81	62/99 (62.6%)	3.5	1/15 (6.7%)	3.8

IPV002	1	IPV–bOPV–bOPV	2-4-6 months	150	117/182 (64.3%)	3.5	5/174 (2.9%)	3.2
	2	IPV–IPV–bOPV	2-4-6 months	166	114/187 (61.0%)	3.5	2/181 (1.1%)	6.3
	3	IPV–IPV–IPV	2-4-6 months	165	119/185 (64.3%)	3.8	3/177 (1.7%)	2.9

‡ Number with available data for both pre-challenge serology and post-challenge shedding index endpoint (SIE).

* Immediately prior to first vaccination.

** 4 weeks after last vaccination, immediately prior to mOPV2 challenge dose.

† Combined across manufacturer group.

### 3.1 Shedding positivity

Fit of the GAM indicates that both NAb titers and post-challenge day are statistically significant predictors of the probability of shedding positivity; the model which includes both days since challenge and NAb titers is significantly better at explaining variation in probability of shedding than one that only includes days since challenge as a factor (p < .0001), which is itself significantly better than the null model (p < .0001). The interaction between NAb titers and study day, however, was not supported by the data (AIC difference of 54 units), indicating similarity in the shape of the relationship between the two variables across post-challenge time point. Comparison with a model which omits the smoothing term in favor of a linear relationship indicates the nonlinear smoothing term is a significant component of the model (p < .0001). The results are supported by repeat testing during the censored-data simulation procedure.

Predictions from the model fit are described in Table 2. The model predicts monotonically decreasing likelihood of shedding with advancing study day for each fixed value of NAb titer, which is consistent with univariate descriptive results (Table 4 in [11], Table 3 in [9]). The relationship between pre-mOPV2-challenge NAb titers and shedding positivity is estimated to be relatively flat for each post-challenge study day, with substantial differences (approximately 5–10% decreases) in estimated shedding positivity limited to a change between NAb levels of 8.5 log_2_ and 10.5 log_2_. This model produces estimated differences in the probability of shedding positivity between NAb LLOQ and ULOQ of 2.7% (0.1%, 5.2%) for Day 7, 6.7% (3.7%, 9.6%) for Day 14, 10.8% (6.7%, 15.0%) for Day 21, 13.4% (8.1%, 18.8%) for Day 28, with a median AUC of the ROC of 0.69 across simulations, compared with 0.67 for the model which includes a factor for study day, but excludes pre-challenge NAbs.

**Table 2:** Model-estimated proportion (95% CI) of mOPV2-challenged vaccinees as a function of day post-challenge and pre-challenge antibody titer level. Proportions are estimated with a generalized additive model (GAM) using a binomial error structure with a logit link.

	Pre-challenge Type 2 Neutralizing Antibody Level (log_2_)				
Day post-challenge	2.5	4.5	6.5	8.5	10.5
7	77.1 (75.3, 78.8)	76.7 (74.9, 78.4)	76.5 (74.7, 78.3)	75.9 (74.1, 77.8)	74.4 (72.6, 76.2)
14	67.4 (65.4, 69.4)	66.4 (64.3, 68.5)	66.0 (63.9, 68.1)	64.6 (62.1, 67.0)	60.7 (58.8, 62.7)
21	56.0 (53.2, 58.8)	54.3 (51.2, 57.4)	53.7 (50.6, 56.7)	51.3 (47.7, 54.9)	45.2 (42.6, 47.7)
28	43.9 (40.0, 47.9)	41.7 (37.5, 45.9)	40.9 (36.8, 44.9)	37.9 (33.3, 42.5)	30.5 (27.4, 33.5)

### 3.2 Magnitude of shedding

**Fig. 1:**
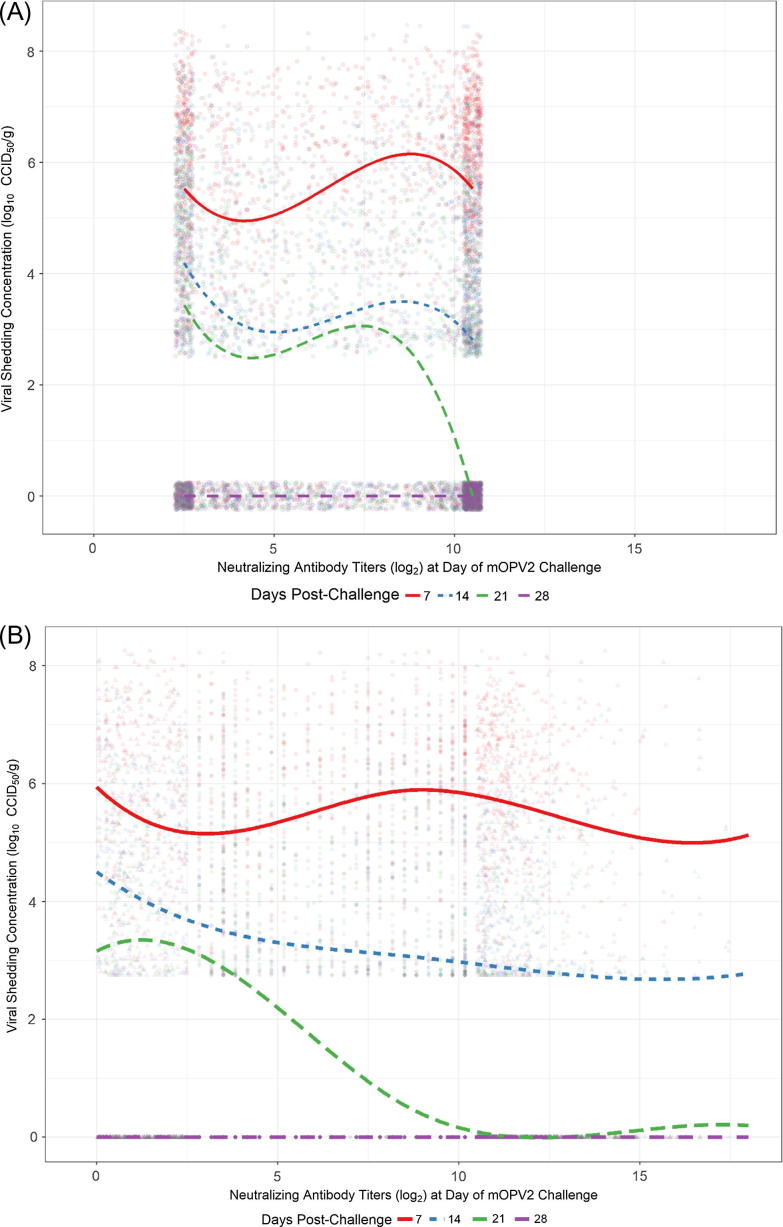
(A) Model-estimated median titer of shed type 2 poliovirus as a function of neutralizing antibody titers pre-mOPV2 challenge and nominal day post-challenge of stool sample collection. Lines indicate regression fit to observed data ignoring censoring at NAb LLOQ (2.5 log_2_) and ULOQ (10.5 log_2_). Observed data points are jittered to convey density. Samples negative for viral shedding are assigned a value of 0. (B) The same as panel A, except data (circles) are not jittered, and values at NAb LLOQ/ULOQ are replaced with one illustrative realization form the censored-data simulation (triangles). Median regression line indicates model fit predictions aggregated across censored data simulations.

Modelling titer of shed virus as a function of NAb titers is depicted in Fig. 1, in which panel A shows (jittered) observed data, and panel B illustrates one example realization from the censored data simulations. Both NAb titers and day post-challenge are statistically significant predictors of titer of shed virus compared with reduced models, as is the interaction between the two variables (all p < .0001). The significantly lower AIC for the model incorporating the smoothing spline indicates that it provides a superior fit to the data compared with a linear model for the median (AIC difference of 80 units).

Not surprisingly, the model predicts monotonically decreasing viral shedding with advancing study day for each fixed value of NAb titer, which is again consistent with univariate descriptive results (Table 4 in [11], Table 3 in [9]). The estimated relationship between NAb titers on the day of mOPV2 challenge and median titer of shed virus changes very little across levels of pre-challenge NAbs on day 7, with an increasingly negative average slope for increasing days from mOPV2 challenge; median viral titer at day 28 is estimated to be 0, across all levels of NAb titer on the day of challenge. The model fit obtained by considering censored values as observed exhibits some similarities (Fig. 1A), but fluctuates more than the model which accommodates the censored predictor values (Fig. 1B).

This model, which explains only 2.5% more variation in weekly shedding than a model which includes only a factor for post-challenge day, produces a slightly higher estimated median viral titer at the NAb ULOQ than the LLOQ for Day 7 (difference of −0.63, [−1.02, −0.23]), with higher median viral titer at the NAb LLOQ compared with the ULOQ for Days 14 (difference of 0.75 [0.33, 1.18]) and 21 (difference of 3.10 [2.21, 3.98]).

### 3.3 Shedding index endpoint

**Fig. 2:**
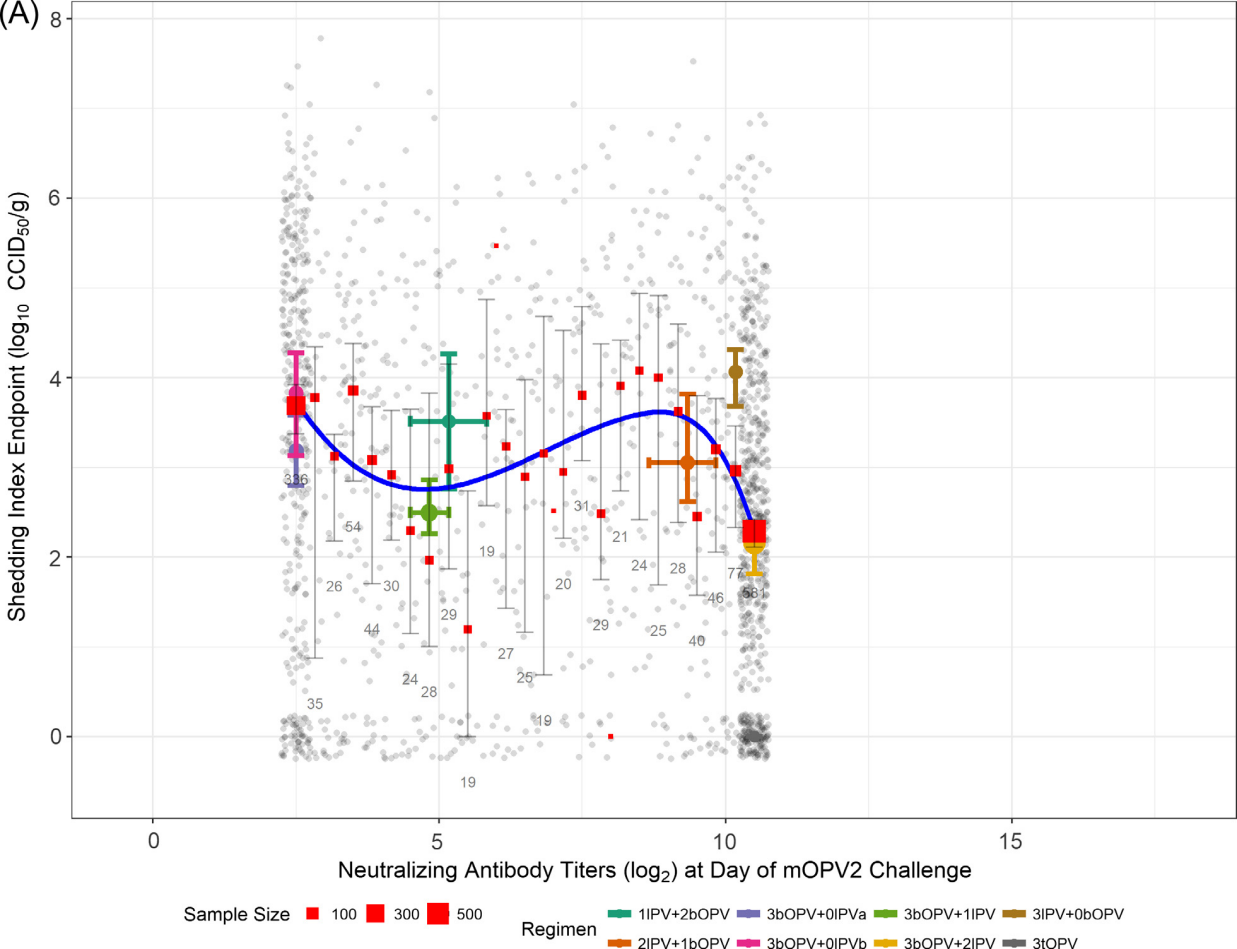

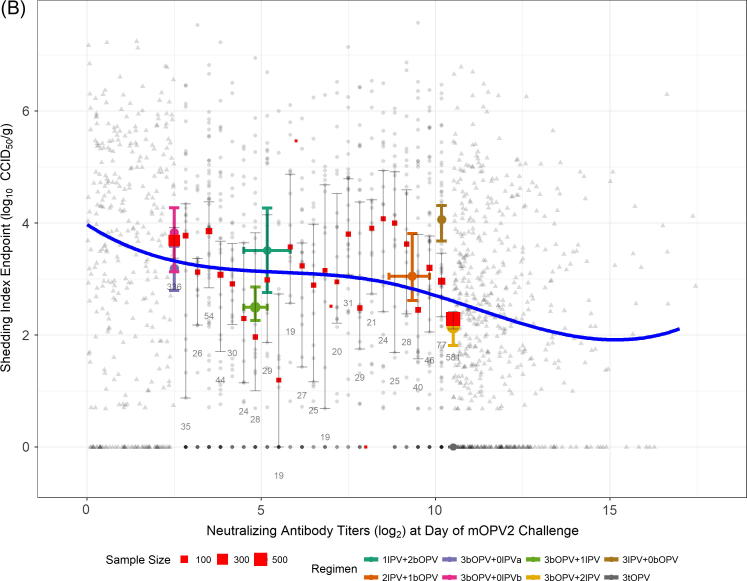
(A) Model-estimated median shedding index endpoint (SIE) of shed poliovirus (type 2) as a function of neutralizing antibody titers pre-mOPV2 challenge. Line indicates regression fit to observed data ignoring censoring at NAb LLOQ (2.5 log_2_) and ULOQ (10.5 log_2_). Observed data points are jittered to convey density. Red boxes and accompanying error bars and numbers indicate the point estimates of median SIE for each discrete level of NAb titer, along with corresponding bootstrap-based 95% confidence intervals, and the number of subjects available for analysis, respectively. Also shown are the medians and accompanying bootstrap-based 95% confidence intervals for both SIE (vertical error bars) and NAb titers (horizontal error bars) for each regimen from the combined studies; symbol size indicates sample size. The IPV001 arm given three doses of tOPV is shown (lower right) to provide context, and was not included in model-fitting procedures. (B) The same as panel A, except data (circles) are not jittered, and values at NAb LLOQ/ULOQ are replaced with one illustrative realization from the censored-data simulation (triangles). Median regression line indicates model fit predictions aggregated across censored data simulations. (For interpretation of the references to colour in this figure legend, the reader is referred to the web version of this article.)

Neutralizing antibody titer is a statistically significant predictor of the SIE (p < .0001 vs null model). The estimated relationship is depicted in Fig. 2, in which panel A shows (jittered) observed data, and panel B illustrates one example realization from the censored data simulations. The significantly lower AIC for the model incorporating the spline basis compared with a linear model for the median indicates nonlinearity in the relationship is an important model feature (AIC difference of 39 units).

The model suggests a detectable, but weak negative relationship exists between extent of viral shedding over 28 days and NAb titers on the day of mOPV2 challenge. The model fit obtained by considering censored values as observed (Fig. 2A) more closely aligns with the fluctuating point estimates of the median taken at each possible value of NAb titers. The model which accommodates the censored predictor values (Fig. 2B) produces a smoother estimate, with a negative slope which increases slightly in magnitude for the highest observed values of NAb titers on day of challenge. This model produces an estimated difference in median (95% CI) SIE between the NAb LLOQ and ULOQ of 0.72 (0.33, 1.11) log_10_ CCID_50_ per gram of stool, and explains a modest 3.0% of total variation in median SIE.

**Fig. 3:**
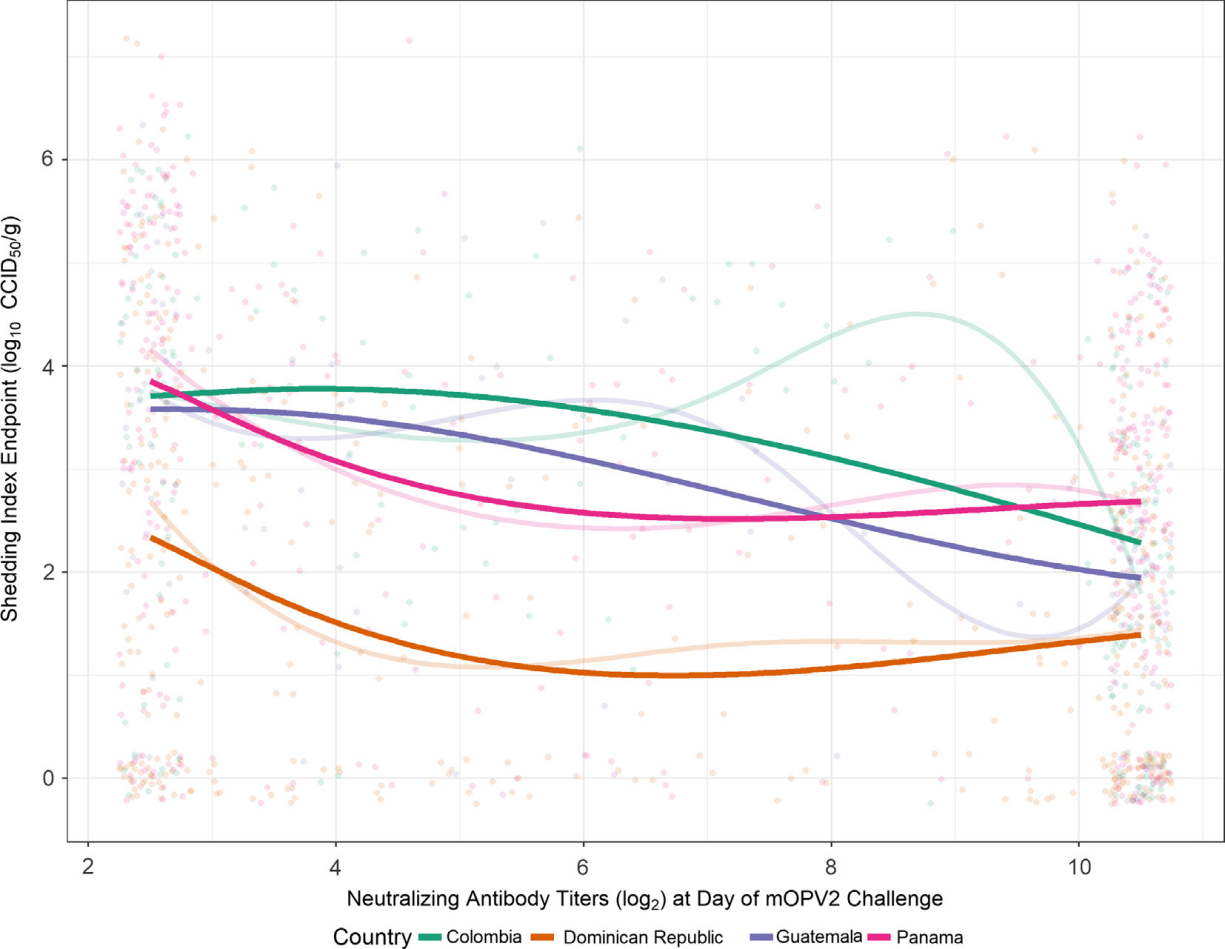
Model-estimated median shedding index endpoint (SIE) of shed poliovirus (type 2) by country as a function of neutralizing antibody titers pre-mOPV2 challenge and nominal day post-challenge of stool sample collection for the subset analysis of IPV001 participants only. Faint line indicates regression fit to observed data ignoring censoring at NAb LLOQ (2.5 log_2_) and ULOQ (10.5 log_2_); bold line indicates aggregated median regression fit predictions from simulation method to account for censoring. Observed data points are jittered to convey density. Figure is limited to observed range of data due to poor performance of the spline fit at the low-sample-size extremes of the NAb range.

The subset analysis of SIE among only IPV001 participants yielded negative estimated relationships of SIE with pre-challenge type 2 serum NAbs which were generally stronger than in the combined-data analysis, evident by larger differences in median SIE across the range of NAb LLOQ and ULOQ (Table 3). The interaction of NAbs with country was statistically significant compared with the nested model which assumed only vertical “shifts” in the relationship (p = .0004), which was itself statistically significantly better than the model which excludes country as a factor entirely (p < .0001). This indicates that there is sufficient evidence to conclude that the relationship between pre-challenge type 2 serum NAbs and SIE differs across country. Fig. 3, however indicates that the relationships, which explain 10.0% of total variation in SIE, are still generally negative, with differences in the convexity or concavity of the regression lines. Specifically, subjects from the Dominican Republic tended to shed lower amounts of virus across levels of pre-challenge type 2 NAbs compared with participants from the other countries, who generally shed similar quantities of virus.

**Table 3:** Estimated differences in median Shedding Index Endpoint (SIE) across the quantifiable range of pre-challenge type 2 neutralizing antibody titers (value at LLOQ minus value at ULOQ) for the subset analysis of IPV001-only participants. Randomization to regimen was balanced across country, and all subjects received three doses of bOPV, and 0, 1, or 2 doses of IPV.

Country (n subjects)	Estimated median difference in SIE (95% CI)
Colombia (191)	1.42 (0.04, 2.81)
Dominican Republic (424)	0.95 (−0.22, 2.11)
Guatemala (111)	1.64 (0.33, 2.95)
Panama (433)	1.17 (0.22, 2.12)

## 4 Discussion

Withdrawal of type 2 OPV and introduction of IPV into routine immunization programs are the first steps in the subsequent removal of all OPV that should eliminate the risks of vaccine-related poliovirus disease and circulation. Improving our understanding of the relationship between development of serum antibodies and mucosal immunity is important to characterize the risk of virus spread in populations immunized only with IPV and plan appropriate response strategies.

Post-vaccination serum neutralizing antibody titers of 4–8 are widely accepted as correlates of protection against paralytic poliomyelitis [19]. The current gold standard surrogate to estimate intestinal immunity is resistance from shedding following an oral challenge [20]. Earlier studies with the Salk vaccine conducted in 1950–60s with the early formulations of IPV indicated a correlation between high levels of serum neutralizing antibodies and reduced fecal virus excretion [21], [22], [23], [24], [25]. However, Onorato et al. [26] demonstrated that even when the humoral response from the currently available IPV was higher than the response provided by those earlier formulations, the higher potency did not provide any gain in intestinal immunity, which was still lower than that obtained with tOPV, and thus questioned the relationship between humoral response and intestinal mucosal immunity. The study also showed that the resistance to intestinal excretion is dependent on the dose of the challenge vaccine and is therefore not absolute. Ogra et al. [27] were the first to report that infants who had received IPV in primary series followed by a dose of tOPV eight months later elicited boosting responses of both serum NAb and secretory IgA against poliovirus. In another study where 67 children vaccinated with tOPV in primary series were followed up for 10 years, waning serum antibody titers were an indicator of decreasing resistance to intestinal excretion [28], yet now it is recognized that intestinal immunity is transient [29], [30]. In a more recent meta-analysis on immunity to polioviruses the authors found indications of potential dependence of shedding post-vaccination with both pre-challenge titers and number of vaccine doses [31]. Mixed or sequential schedules of IPV and tOPV have been studied extensively, and patterns of humoral and intestinal immunity in infants receiving a range of primary schedules of IPV and tOPV in different dose combinations have been described [9], [10], [11], [32], [33], [34], [35], [36], [37]. Studies evaluating IPV and bOPV in primary series are limited in number, and the two trials reported here [9], [11] are the only ones thus far that describe a unique and detailed type 2 shedding pattern in terms of titers, prevalence and duration of virus excretion in the context of an mOPV2 challenge. This situation closely simulates the likely post-eradication schedule of routine immunization and type 2 virus re-introduction scenario.

We found a weak negative relationship between humoral antibodies and excretion of shed virus. The estimated probability of shedding for each of 4 weeks post-mOPV2 challenge declines gradually with increasing NAb titers, but the observed difference in shedding probability across the entire measured range of NAb titers at Day 28 peaks at approximately 13%, a relative decline of approximately 30% from the estimated probability at NAb LLOQ. In contrast, the estimated median titer of shed virus at 3-week post-challenge exhibits substantial variation across NAb titers – a decline of approximately 93% from NAb LLOQ to ULOQ. This difference in median viral shedding across the extremes of pre-challenge titer is significantly lower on Day 14, and disappears by Day 28, where median shedding has dropped to 0 (more non-shedders than shedders) for all levels of pre-challenge NAb.

The SIE, the combination measure of duration and quantity of shed virus, is estimated to decline gradually with increasing pre-challenge NAb titer. Across the range of pre-challenge NAb titers, the predicted relative decline in SIE is estimated to be approximately 22%. Results from IPV001 Group 2 (bOPV only) vs Group 5 (bOPV plus two IPV) [11] and Fig. 2, indicate a larger difference in estimated median SIE at these NAb levels; the additional data incorporated in this regression model, and the use of NAbs as the sole predictor rather than regimen serve to attenuate the estimated difference.

While the relationships estimated here are detectable, consistent, and statistically significant, they do not account for a substantial amount of variability in shedding characteristics. Measures of model fit, including the ROC AUC for shedding positivity and in particular the proportion of variation explained by median regression for weekly viral titers and SIE, indicate some significant unexplained variation in viral shedding remains beyond that explained by pre-challenge NAb titers. Heterotypic protection from bOPV could not be rigorously assessed from these combined data. However, previously published results from IPV002 showed subjects given three doses of IPV but no bOPV shed virus in greater quantities than those given fewer IPV doses along with bOPV [9], suggesting that cross-protection may be one factor inducing variation in viral shedding outcomes [38]. The impact of heterotypic intestinal immunity may have served to attenuate the estimated relationship between type 2 serum NAbs and viral shedding (for example, IPV002 subjects receiving three IPV doses but no bOPV had very high serum NAb levels, and also had higher median SIE than any other group). Other confounding variables may exist. For example, subjects with greater humoral immune responses may be naturally inclined to have lower intestinal immune responses through features of the individual’s immune system or epidemiological factors, although we consider this unlikely. Additional features of individual immunity, however, were not measured in these studies. A subset analysis was undertaken to estimate and control for the role of potential differences in passive exposure to circulating type 2 virus that would not be evident in those with pre-challenge NAbs near the ULOQ, but would be manifest in greater intestinal immunity (lower viral shedding). This subset analysis indicates that while differences in viral shedding were seen across countries, the differences in the shape of the relationship between the two variables within each country were small (albeit statistically significant), and the overall weak negative relationship of the two variables holds. The primary difference between the main and subset analyses is that the combined-data analysis serves to attenuate the estimated relationship between the two variables, potentially through the lack of heterotypic intestinal immunity conferred by the IPV002 IPV-only regimen.

In comparison with viral shedding results from the bOPV–IPV regimens considered here, subjects from IPV001 who received three doses of tOPV (without IPV) shed very low quantities of type 2 virus (Fig. 2 and [11]). These subjects also had very high levels of pre-challenge NAbs. Mucosal intestinal immunity to type 2 induced by multiple doses of tOPV is clearly superior to that provided by the bOPV–IPV regimens considered here [39]. The magnitude of this difference in viral shedding may be expected to provide a substantial reduction in virus transmission compared with that provided by bOPV–IPV regimens, although only limited data are available to directly link dynamics of viral shedding to transmission [40], [41]. Regardless, our data indicate that very high pre-challenge type 2 NAb titers are insufficient in themselves to substantially impact viral shedding.

Overall, the weak relationship we found in this exploratory analysis serves to support the potential distinctiveness of humoral and mucosal immune responses in the new endgame mixed or sequential bOPV-IPV schedules the and underscores the importance for further exploration of the dynamics of intestinal mucosal immunity in such schedules and settings.

One limitation of our analysis is that the SIE is an imperfect endpoint because it is a mean of potentially censored values. Regression for the median SIE was employed to mitigate this weakness. The SIE, however, remains a meaningful endpoint that captures both the extent and duration of viral shedding in a single index and is thus important from an epidemiological standpoint to assess risk of viral shedding and transmission. The heterogeneity of different schedules, populations and social and epidemiological factors in the countries where the trials were conducted also introduced uncontrolled variation in the overall assessment. The use of censored NAb (predictor) values is a weakness of the regression modeling. The use of a simulation method to assess the sensitivity of results to this censoring serves to mitigate this weakness, although the model for censored values chosen here is a simple one, and other choices may also be reasonable. Other unmeasured and unknown confounding variables may exist, but the most important known potential confounders, heterotypic intestinal immunity conferred by bOPV administration and potential differences in exposure to circulating type 2 virus across the region, have been investigated, and it appears that neither is associated with artificially strengthening the observed relationship between pre-challenge type 2 serum NAbs and measures of intestinal immunity based on viral shedding.

## 5 Conclusions

High serum NAb titers against poliovirus type 2 at the time of mOPV2 challenge among IPV vaccinated infants are only weakly associated with lower viral shedding. Thus, NAb titers at 4 weeks post-final vaccination are an insufficient correlate of intestinal immunity. The impact of the magnitude of reduction in shedding associated with high NAb titers remains unclear. Research directed toward defining stronger correlates of polio intestinal immunity should facilitate better understanding of population vulnerability to poliovirus transmission in the final phases of polio eradication.

## 6 Conflicts of interest and funding statement

This study was funded by the Bill & Melinda Gates Foundation (BMGF). ASB and JFM are full-time employees of the BMGF and contributed to the study design, data interpretation and writing of the manuscript. Other authors confirm they have no conflicts of interest to declare.

All authors contributed to the design and/or conduct of the studies, and the elaboration of this manuscript, and agree to the submission to the journal.
